# Mr. Spencer's Case of Idiosyncracy

**Published:** 1809-06

**Authors:** 


### *To the Editors of the Medical and Physical Journal.*

GENTLEMEN,

IN Mr. Royston's interesting Remarks "on a Medical Topography," a fact is recorded of a man who could not bear the effluvia arising from the pulverization of ipecacuanha, without suffering from it most severely. The words indeed are, "that he was unable to remain in a house where the pulverization of that vegetable was performing." Seeing

Seeing this, has encouraged me briefly to state to you, for the curious in medicine, a case of similar idiosyncracy.

An apprentice of mine, naturally healthful, and of an active disposition, is invariably affected with a most distressing and protracted sneezing on the most careful dispensing of the smallest quantity of ipecacuanha. A more continued application of it, such, for instance, as happens in the preparation of the compound powder, is followed with dyspnœa, cough, and spitting of blood. Having occasion, some time ago, to compound the medicine for several days together, he became seriously affected by it, in the way just stated; and he has not enjoyed full health since. I have hitherto encouraged him, by holding out to him, that a frequent use of it might perhaps remove the susceptibility to impression; on the contrary, however, his symptoms become every day more aggravated on its use, and, in his present disposition, there is reason to fear that it may do him an irreparable injury. Indeed, it has already evidently produced "a disposition to asthma, and an aptitude for pulmonary ailment," which he had not used to possess.

This fact is of serious consequence to the sufferer, connected as he is with medicine, and called upon, as he frequently must be, to prepare as a remedy for others, the very doing of which is to himself so very deleterious. It becomes, therefore, a question of great interest to him, if it extend not to the profession at large. How is this morbid sensibility to be removed?

Another fact, connected with this history, shewing still more forcibly the poisonous influence which this drug has over him, ought to be mentioned, and especially as it is of practical consideration. Last autumn he had the jaundice; it was recommended to him to take an emetic; he took the ipecac. wine with a common portion of antimony. The effect was highly alarming. I was called to him from an adjoining room, and found him in the greatest agony. He had a wild and frantic look, his whole countenance was livid, and his lips in particular were black and protuberant; but what most distrest him was an intolerant itching all over his body, in the impatience of which he had flung off his clothes, and seemed ready to tear himself to pieces. The character of his countenance being thus altered, I at first thought might be owing to the mere act of vomiting; and it naturally occurred to me, that the itching was in some degree attributable to his complaint. But he assured me to the contrary, and for the first time informed informed me, that an emetic which he formerly took, affected him exactly in the same manner. After an opiate he became easier, and in the morning arose free from complaint; as far at least as regarded the emetic.

